# Neutrophil-driven tumor inflammation in breast cancer: Pathways and therapeutic implications: A narrative review

**DOI:** 10.1097/MD.0000000000043024

**Published:** 2025-06-20

**Authors:** Emmanuel Ifeanyi Obeagu

**Affiliations:** a Department of Biomedical and Laboratory Science, Africa University, Mutare, Zimbabwe.

**Keywords:** breast cancer, neutrophils, therapeutic implications, tumor inflammation, tumor microenvironment

## Abstract

Neutrophils, a key component of the innate immune system, have emerged as significant players in the tumor microenvironment (TME) of breast cancer. Traditionally recognized for their role in defending against infections, neutrophils in cancer contribute to both tumor progression and suppression, depending on their activation state and the signals they receive. Neutrophil-driven inflammation within the TME is critical in promoting tumor growth, metastasis, and resistance to treatment. This review explores the complex role of neutrophils in breast cancer, emphasizing the mechanisms through which they influence tumor inflammation, including their interactions with other immune cells and the signaling pathways involved. Neutrophils can either support tumor progression by releasing pro-inflammatory cytokines, enzymes, and reactive oxygen species, or contribute to antitumor responses by directly killing cancer cells and stimulating adaptive immunity. The balance between these opposing functions is heavily influenced by the TME’s cytokine milieu and neutrophil polarization. Key signaling pathways, such as the CXCR2-CXCL8 axis and interactions with tumor-associated macrophages, regulate neutrophil recruitment and activation in the TME, enhancing inflammation and tumorigenesis. Additionally, neutrophils’ ability to form neutrophil extracellular traps further complicates their role, potentially promoting metastasis by trapping circulating tumor cells.

## 1. Introduction

Breast cancer is one of the most common malignancies affecting women worldwide and continues to be a leading cause of cancer-related mortality.^[1,2]^ Over the past few decades, significant strides have been made in understanding its molecular and cellular mechanisms, which has led to advancements in treatment strategies. Despite these improvements, a large proportion of breast cancer patients still experience disease progression and relapse. The TME plays a crucial role in the pathogenesis of breast cancer, influencing tumor behavior, immune evasion, metastasis, and treatment resistance. Among the various immune cells that inhabit the TME, neutrophils have recently garnered attention due to their significant involvement in both promoting and inhibiting tumor progression.^[3,4]^ Traditionally, neutrophils were considered to be part of the innate immune system primarily responsible for defending the body against bacterial infections. They act as the first line of defense by responding rapidly to inflammatory signals, phagocytosing pathogens, and releasing antimicrobial molecules. However, recent research has revealed that neutrophils can adopt a more complex role in cancer biology, particularly in their involvement in the TME. Their recruitment and activation in tumors are influenced by various factors, including cytokines and growth factors, creating a highly dynamic and often pro-inflammatory environment. While neutrophils are crucial for immune defense, their role in cancer is paradoxical, as they can either support tumor progression or contribute to tumor suppression.^[5]^

One of the primary functions of neutrophils in the TME is to modulate inflammation. Tumor-associated inflammation is now recognized as a hallmark of cancer progression. Chronic inflammation within the TME can create a conducive environment for tumor cells to proliferate, invade surrounding tissues, and acquire resistance to immune surveillance and therapy. Neutrophils contribute to tumor inflammation by secreting a wide range of cytokines, proteases, and reactive oxygen species (ROS) that can influence tumor growth and metastasis. These inflammatory mediators promote angiogenesis, enhance tumor cell survival, and facilitate the degradation of extracellular matrix components, thereby supporting tumor invasion and metastasis.^[6,7]^ In addition to their role in promoting inflammation, neutrophils are also capable of inducing antitumor responses. Under certain conditions, neutrophils can polarize into a pro-inflammatory, tumor-suppressing phenotype. In these cases, they can directly kill cancer cells by releasing cytotoxic granules containing enzymes such as elastase and proteinase 3. Moreover, neutrophils can activate adaptive immune responses by interacting with dendritic cells and T cells, fostering an environment where tumor-specific immune responses are more robust. Thus, neutrophils exhibit a dual role in breast cancer, depending on their activation state, the presence of inflammatory mediators, and their interactions with other immune cells within the TME.^[8]^

The complex interplay between neutrophils and other immune cells, including macrophages, T cells, and tumor-associated fibroblasts, adds further complexity to the role of neutrophils in cancer. tumor-associated macrophages, for example, can influence neutrophil function by releasing cytokines like G-CSF and GM-CSF, which recruit neutrophils to the tumor site. Neutrophils, in turn, can influence macrophage polarization, either promoting a pro-inflammatory M1 phenotype or an anti-inflammatory M2 phenotype. This cross-talk between neutrophils and other immune cells is essential in shaping the immune landscape of the TME and determining the overall tumor-promoting or tumor-suppressing effects of neutrophils.^[9,10]^ In breast cancer, neutrophils have been implicated in the development of resistance to conventional therapies such as chemotherapy, radiation, and targeted therapies. By producing inflammatory mediators, neutrophils can create a shield around the tumor, protecting cancer cells from the effects of treatment. Moreover, neutrophils are involved in the process of metastasis, particularly through the formation of neutrophil extracellular traps (NETs). NETs are web-like structures composed of chromatin and antimicrobial proteins that neutrophils release in response to certain stimuli. While NETs play a role in trapping pathogens, they can also entrap circulating tumor cells, providing them with a platform to adhere to endothelial cells, facilitating tumor cell extravasation and the establishment of secondary tumors.^[11–13]^ Recent advancements in cancer immunotherapy have led to renewed interest in modulating immune cell activity to treat breast cancer. Given the prominent role of neutrophils in the TME, there is growing interest in exploring therapeutic strategies that target neutrophil-driven inflammation to improve treatment outcomes. These strategies include inhibiting neutrophil recruitment to the tumor site, blocking neutrophil activation, and modulating neutrophil polarization to enhance their antitumor effects. Additionally, targeting NET formation has shown promise as a potential therapeutic approach in preventing metastasis and improving responses to chemotherapy and immunotherapy.

### 1.1. Aim

The aim of this review is to explore the role of neutrophils in driving tumor inflammation in breast cancer, emphasizing the key pathways involved in their recruitment, activation, and interaction with the TME.

### 1.2. Rationale

A significant challenge in treating breast cancer is the TME, which is a highly dynamic and immunosuppressive setting that fosters tumor growth, metastasis, and resistance to therapy. Among the diverse immune cell populations within the TME, neutrophils have emerged as critical players in tumor progression. While neutrophils are traditionally associated with the body’s defense against infections, their role in cancer has become increasingly recognized for their dual functionality. In breast cancer, neutrophils can promote inflammation that supports tumor growth, angiogenesis, and metastasis, or alternatively, they can contribute to antitumor immunity. However, the balance between these opposing roles is often skewed toward pro-tumorigenic activities, leading to the exacerbation of cancer progression and treatment resistance. The rationale for focusing on neutrophil-driven inflammation in breast cancer stems from the growing body of evidence that highlights the central role of neutrophils in shaping the TME. Neutrophils are recruited to tumors through a variety of chemokines and cytokines, and once there, they can promote the secretion of matrix metalloproteinases, ROS, and pro-inflammatory cytokines that facilitate tumor progression. Furthermore, recent studies have shown that neutrophils contribute to immune evasion by suppressing T-cell responses and enhancing the angiogenic capacity of the tumor. This inflammation-driven mechanism of tumor progression makes neutrophils an attractive target for therapeutic intervention. Understanding the pathways that govern neutrophil recruitment, activation, and polarization in the TME is crucial for designing effective therapies that can either reprogram neutrophils to fight the tumor or inhibit their pro-tumorigenic effects.

Given the multifaceted role of neutrophils in both promoting and inhibiting tumor growth, targeted therapies that specifically modulate neutrophil activity could offer a novel strategy for breast cancer treatment. However, the challenges associated with targeting neutrophil-driven inflammation are significant, as neutrophils are integral to the body’s immune response and their dysregulation can lead to unintended immune suppression or inflammatory side effects. Therefore, a careful and precise approach is needed to develop therapies that specifically target the detrimental effects of neutrophils without compromising overall immune function. The rationale for this review is to synthesize the current understanding of neutrophil-driven inflammation in breast cancer and explore how therapeutic strategies could be tailored to exploit these cells’ role in cancer progression while minimizing potential risks. This research is pivotal in advancing breast cancer treatment and improving patient survival rates by targeting one of the most critical and dynamic components of the TME.

## 2. Review methodology

### 2.1. Literature search strategy

A comprehensive literature search was conducted across several academic databases, including PubMed, Scopus, and Web of Science, to identify relevant studies on neutrophil-driven inflammation in breast cancer. The search was carried out using a combination of keywords such as “neutrophils,” “tumor inflammation,” “breast cancer,” “tumor microenvironment,” “immune evasion,” and “therapeutic implications.” Boolean operators (AND, OR) were used to combine these terms, ensuring a broad scope of results. Only peer-reviewed journal articles, clinical studies, preclinical models, and review papers were included in the search.

### 2.2. Inclusion and exclusion criteria

The inclusion criteria were

- Studies focused on the role of neutrophils in breast cancer.
- Research that investigated neutrophil-driven inflammation and its impact on tumor progression, metastasis, and immune evasion.
- Studies examining neutrophil-targeted therapeutic approaches in breast cancer treatment.
- Articles published in English.

Exclusion criteria included

- Studies unrelated to breast cancer or neutrophils.
- Articles that did not provide empirical data or relevant insights into neutrophil-mediated tumor inflammation.
- Case reports and editorials, as they were not considered sufficient for this review’s aim.

### 2.3. Ethical approval

Not applicable as this is a narrative review

### 2.4. Identification of gaps in current knowledge

During the review process, areas where further research is needed were identified, particularly in the understanding of neutrophil polarization in the tumor microenvironment and the long-term effects of neutrophil-targeted therapies. The limitations of current therapeutic strategies, including potential side effects and the risk of immune suppression, were also highlighted.

### 2.5. Neutrophils and tumor inflammation in breast cancer

Neutrophils, a key component of the innate immune system, have long been recognized for their role in the body’s defense against pathogens. However, their involvement in cancer biology, particularly in breast cancer, has become an area of increasing research interest. In the context of breast cancer, neutrophils contribute significantly to tumor inflammation, influencing both tumor progression and suppression, depending on their activation state and the TME. These immune cells infiltrate the TME in response to a variety of signals, where they can exacerbate inflammation, promote tumor growth, or play a role in immune defense.^[14]^ One of the central functions of neutrophils in cancer is their ability to drive inflammation within the TME. Tumor inflammation is now considered one of the hallmarks of cancer, promoting various processes such as tumor growth, metastasis, and resistance to treatment. Neutrophils contribute to this by releasing a range of pro-inflammatory cytokines, proteases, and ROS that can create a favorable environment for tumor cells to proliferate and invade surrounding tissues. For example, neutrophils secrete interleukins (IL-1, IL-6, and IL-8) and tumor necrosis factor-alpha (TNF-α), which can stimulate tumor cell growth and angiogenesis, a process that ensures a continuous blood supply to the tumor. These inflammatory mediators can also degrade extracellular matrix components, facilitating tumor invasion and metastasis. In this way, neutrophils contribute to an inflammatory microenvironment that accelerates tumor progression and hinders immune surveillance.^[14,15]^

However, neutrophils in breast cancer can also play a protective role by mounting an antitumor immune response. Under certain conditions, neutrophils can polarize into a pro-inflammatory phenotype that exhibits cytotoxic activity against cancer cells. Neutrophils can kill tumor cells directly by releasing cytotoxic granules containing enzymes such as elastase and myeloperoxidase. Additionally, neutrophils can promote the activation of adaptive immune responses. For instance, they can influence dendritic cells to enhance T-cell activation and shape the adaptive immune response against tumor cells. Neutrophils have also been shown to induce a shift in macrophage polarization, which can influence the TME in a manner that may support tumor suppression rather than progression. This dual nature of neutrophils – both promoting and inhibiting tumor growth – highlights the complexity of their role in breast cancer.^[16,17]^ Despite their potential for tumor suppression, the ability of neutrophils to exacerbate tumor inflammation and contribute to resistance to therapies presents significant challenges in breast cancer treatment. One of the mechanisms through which neutrophils contribute to therapy resistance is by forming NETs. NETs are web-like structures composed of chromatin and antimicrobial proteins released by activated neutrophils. While NETs help trap pathogens, they can also ensnare circulating tumor cells, providing a platform for tumor cells to adhere to blood vessel walls and potentially metastasize to distant organs. This process is particularly concerning in breast cancer, where metastasis is often the primary cause of mortality. The presence of NETs can also interfere with the effectiveness of treatments such as chemotherapy, immunotherapy, and radiation, as they may shield tumor cells from the immune system and therapeutic agents.^[18,19]^

### 2.6. Key pathways involved in neutrophil-driven tumor inflammation

Neutrophils are central players in the TME, contributing to tumor inflammation and influencing cancer progression through various key pathways. These pathways not only mediate neutrophil recruitment and activation but also shape the inflammatory milieu within the TME, which can either promote or inhibit tumor growth. Below are some of the primary pathways involved in neutrophil-driven tumor inflammation:

#### 2.6.1. CXCL8/CXCR2 axis

The CXCL8 (IL-8)/CXCR2 axis is one of the most well-characterized pathways mediating neutrophil recruitment to the TME. CXCL8 is a potent chemokine produced by tumor cells and stromal cells in response to various inflammatory stimuli. It binds to the CXCR2 receptor on neutrophils, triggering their migration toward the tumor site. Once in the TME, neutrophils can release additional cytokines, proteases, and ROS, further enhancing the inflammatory environment. Inhibition of the CXCL8/CXCR2 axis has been shown to reduce neutrophil infiltration, decrease tumor-promoting inflammation, and inhibit tumor metastasis in preclinical models. Consequently, this pathway represents a potential therapeutic target to limit neutrophil-driven inflammation in cancer.^[20,21]^

#### 2.6.2. NF-κB signaling

The NF-κB signaling pathway is crucial for neutrophil activation and inflammation. Upon activation by inflammatory mediators, such as cytokines, chemokines, or pathogen-associated molecular patterns, NF-κB translocates to the nucleus and initiates the transcription of pro-inflammatory genes. This includes the production of cytokines like TNF-α, IL-1β, and IL-6, which enhance neutrophil recruitment and activation. In the context of cancer, NF-κB signaling in neutrophils contributes to the secretion of factors that support tumor growth, angiogenesis, and metastasis. Targeting NF-κB signaling in neutrophils could dampen the inflammatory response in the TME and hinder the progression of cancer.^[22,23]^

#### 2.6.3. Neutrophil extracellular traps formation

NETs are networks of extracellular fibers composed of chromatin and antimicrobial proteins that neutrophils release in response to inflammatory signals. NETs serve to trap and neutralize pathogens, but in cancer, they play a role in promoting tumor metastasis. NETs facilitate the adhesion of circulating tumor cells to the endothelial lining of blood vessels, aiding in their extravasation and colonization of distant organs. Furthermore, NETs can protect tumor cells from immune surveillance and chemotherapy by shielding them from immune cells and therapeutic agents. Targeting NET formation or degrading existing NETs has been proposed as a strategy to prevent metastasis and enhance the efficacy of cancer treatments.^[19,24]^

#### 2.6.4. ROS production

Neutrophils produce ROS as part of their antimicrobial defense. However, in the TME, excessive ROS production can contribute to inflammation, DNA damage, and tumor progression. ROS can modify proteins, lipids, and DNA within the tumor microenvironment, creating a pro-tumorigenic inflammatory state. ROS also promote angiogenesis by stimulating the secretion of vascular endothelial growth factor (VEGF) from tumor cells and stromal cells. In addition, ROS can enhance the resistance of tumor cells to chemotherapy and radiation by promoting genomic instability and activating survival pathways. Controlling ROS production in neutrophils within the TME could therefore limit tumor inflammation and potentially increase the efficacy of cancer treatments.^[25,26]^

#### 2.6.5. TGF-β signaling

Transforming growth factor-beta (TGF-β) is a key cytokine in the regulation of immune responses, including neutrophil function. In the TME, TGF-β is often elevated and contributes to the polarization of neutrophils toward a pro-tumor (N2) phenotype, which promotes immunosuppressive and tumor-supporting functions. TGF-β signaling in neutrophils can enhance their ability to secrete matrix metalloproteinases (MMPs), which degrade extracellular matrix components and facilitate tumor cell migration and metastasis. Additionally, TGF-β can suppress the antitumor activities of other immune cells, such as cytotoxic T lymphocytes and natural killer cells, further promoting tumor survival. Targeting TGF-β signaling in neutrophils may help shift their phenotype toward an antitumor (N1) state and reduce tumor progression.^[27,28]^

#### 2.6.6. IL-17 pathway

The IL-17 pathway plays a critical role in the recruitment and activation of neutrophils in inflammation. IL-17 is produced by various immune cells, including T-helper 17 (Th17) cells, and stimulates the production of cytokines such as IL-8, which are involved in neutrophil recruitment to sites of infection or injury. In breast cancer, the IL-17/IL-17 receptor axis has been shown to promote neutrophil infiltration into the TME and contribute to tumor growth and metastasis. Elevated IL-17 levels are often associated with poor prognosis in breast cancer patients, and targeting this pathway could reduce neutrophil-driven inflammation and potentially inhibit tumor progression.^[29,30]^

#### 2.6.7. CD40/CD40L signaling

The CD40/CD40L interaction is an important co-stimulatory pathway in immune responses. In the context of neutrophils, the CD40/CD40L signaling axis influences their activation and the inflammatory responses within the TME. CD40L, expressed on activated T cells and other immune cells, interacts with CD40 on neutrophils, leading to the release of pro-inflammatory cytokines and chemokines. This pathway also supports the creation of a tumor-promoting inflammatory environment, driving tumor progression and metastasis. Therapeutic strategies that block the CD40/CD40L pathway may help suppress neutrophil-driven inflammation in tumors and enhance the immune response to cancer.^[31,32]^

### 2.7. Critical evaluation of conflicting findings in neutrophil biology and tumor progression

The role of neutrophils in cancer progression – particularly in breast cancer – has stirred considerable scientific discourse, marked by both consensus and contention. While emerging literature increasingly portrays neutrophils as pivotal mediators of tumor inflammation, immune modulation, and metastasis, contrasting studies have highlighted their potential anti-tumoral functions, resulting in a complex and sometimes contradictory landscape.^[33]^ One of the central points of conflict lies in the phenotypic plasticity of neutrophils, especially the distinction between the pro-tumoral N2 and anti-tumoral N1 subtypes. While studies such as Fridlender *et al* introduced this binary classification, asserting that tumor-derived TGF-β skews neutrophils toward the N2 phenotype, subsequent research questioned the simplicity of this polarization. For instance, neutrophils have been observed to express both pro- and anti-inflammatory markers simultaneously, suggesting a continuum of activation states rather than a strict N1/N2 dichotomy. This has led to skepticism regarding the applicability of such categorization across different tumor types and stages.^[34]^ Another area of divergence is the prognostic value of the neutrophil-to-lymphocyte ratio (NLR). Multiple studies have established a high NLR as an indicator of poor prognosis in breast cancer patients. However, others have shown no consistent association, particularly in triple-negative breast cancer, raising questions about the context-specific relevance of this marker. These discrepancies may arise from methodological differences – such as variations in cutoff thresholds, timing of measurement, or treatment regimens – which complicate the interpretation and generalizability of findings.^[35]^

Furthermore, NETs are another contentious topic. On 1 hand, NETs have been implicated in enhancing metastatic potential by trapping circulating tumor cells and promoting a pre-metastatic niche. On the other, some researchers argue that NET formation is primarily a response to systemic inflammation or infection and may not be inherently tumor-supportive. Intriguingly, a few animal models have even demonstrated tumor-inhibitory effects following NET induction, further muddying the waters. This suggests that the context in which NETs form – whether acute vs chronic inflammation – may determine their functional consequence in tumor biology.^[36]^ Adding another layer of complexity is the temporal role of neutrophils. Some findings indicate that neutrophils may exhibit antitumor behavior in early tumor development by promoting cytotoxicity and enhancing immune recognition, only to shift toward a tumor-promoting role in later stages due to prolonged exposure to tumor-derived factors. This dynamic behavior challenges static interpretations of neutrophil function and calls for longitudinal studies that trace neutrophil behavior throughout the entire course of tumor progression.^[37]^ Moreover, discrepancies in experimental models contribute significantly to conflicting conclusions. In vitro studies often fail to recapitulate the intricate cellular cross-talk and spatial organization present in the tumor microenvironment. Similarly, while murine models have offered vital mechanistic insights, species-specific differences in neutrophil biology – such as lifespan, surface markers, and receptor expression – limit the translatability of findings to human pathology.^[38]^ There is a growing recognition that neutrophil heterogeneity is influenced not only by the tumor milieu but also by systemic factors such as microbiota, metabolic status, and host genetics. This underappreciated complexity suggests that the so-called “tumor-associated neutrophil” may not be a uniform population but rather a diverse ensemble of cells with context-dependent behaviors – making unified therapeutic targeting inherently challenging.^[39]^

### 2.8. Therapeutic implications: targeting neutrophil-driven inflammation

Neutrophil-driven inflammation plays a pivotal role in tumor progression, metastasis, and treatment resistance, particularly in breast cancer. While neutrophils are essential components of the immune response, their involvement in chronic inflammation within the TME can fuel cancer progression. Therefore, targeting neutrophil-mediated inflammation presents a promising therapeutic strategy to disrupt the inflammatory milieu of the TME and enhance the effectiveness of conventional and immunotherapies. Several potential therapeutic approaches focus on modulating neutrophil activity and preventing their harmful effects on cancer progression.

#### 2.8.1. Inhibition of neutrophil recruitment

One of the most direct strategies for targeting neutrophil-driven inflammation in breast cancer is inhibiting their recruitment to the TME. Chemokine receptors, such as CXCR2, play a crucial role in neutrophil migration to tumor sites, driven by chemokines like CXCL8 (IL-8). Several therapeutic agents have been developed to target this axis, including CXCR2 antagonists and CXCL8 inhibitors. These inhibitors can block neutrophil infiltration, thereby reducing the inflammatory environment that supports tumor growth and metastasis. For instance, in preclinical models, the use of CXCR2 antagonists has led to decreased neutrophil accumulation, reduced tumor progression, and improved response to chemotherapy. This approach can be particularly beneficial in solid tumors like breast cancer, where neutrophils contribute significantly to tumor-related inflammation and angiogenesis.^[40]^

#### 2.8.2. Modulating neutrophil polarization

Neutrophils can adopt different functional phenotypes depending on the signals they receive in the TME. While N1 neutrophils are anti-tumorigenic and promote immune responses against cancer, N2 neutrophils are pro-tumorigenic, facilitating tumor growth and metastasis through the release of cytokines, proteases, and ROS. The polarization of neutrophils toward an N1 phenotype could, therefore, offer a potential therapeutic strategy. TGF-β inhibitors and NF-κB inhibitors are examples of therapeutic agents that could shift neutrophil polarization from the N2 (pro-tumor) to the N1 (antitumor) state. For instance, blocking TGF-β signaling has been shown to reduce the immunosuppressive effects of neutrophils in the TME, improving the efficacy of immune checkpoint inhibitors (ICIs) and chemotherapy. This strategy may enhance antitumor immunity and help reshape the TME to be less supportive of cancer cell survival.^[41]^

#### 2.8.3. Inhibition of neutrophil extracellular trap formation

NETs are structures composed of DNA and antimicrobial proteins released by activated neutrophils, which can contribute to cancer metastasis by providing a scaffold for circulating tumor cells. NETs facilitate tumor cell adhesion to endothelial cells, promoting extravasation and the formation of secondary tumors. Inhibition of NET formation could reduce metastasis and enhance the effectiveness of anti-cancer therapies. Drugs that target histone deacetylases (HDACs) or DNase (which breaks down NETs) are being investigated to prevent NET formation and thereby reduce the metastatic potential of tumors. Preclinical studies have shown that using DNase or HDAC inhibitors can effectively degrade NETs, reducing metastasis and improving therapeutic outcomes in breast cancer models.^[24]^

#### 2.8.4. Targeting neutrophil-derived ROS

Excessive production of ROS by neutrophils contributes to DNA damage, immunosuppression, and tumor progression. ROS also aid in the survival of cancer cells by promoting angiogenesis and resistance to chemotherapy. Antioxidant-based therapies, such as the use of N-acetylcysteine (NAC) or superoxide dismutase mimetics, aim to reduce ROS levels in the TME, thus mitigating neutrophil-driven tumor inflammation. In breast cancer, the use of ROS inhibitors in combination with chemotherapy or immunotherapy has shown promise in preclinical trials by enhancing the cytotoxic effects of drugs and reducing resistance mechanisms. By controlling ROS production, these therapies may improve the therapeutic response and inhibit the pro-tumorigenic effects of neutrophils.^[42]^

#### 2.8.5. Blocking the IL-17 pathway

The IL-17 pathway is a key mediator of neutrophil recruitment and activation in cancer. Elevated IL-17 levels in the TME are associated with poor prognosis in several cancers, including breast cancer. IL-17 promotes the release of chemokines such as CXCL8, leading to neutrophil infiltration, and enhances the production of pro-inflammatory cytokines that support tumor growth. Targeting the IL-17 receptor with monoclonal antibodies or small molecule inhibitors could reduce neutrophil-mediated inflammation and tumor progression. Clinical trials exploring IL-17 inhibitors are ongoing, and promising results have been observed in preclinical models where blocking IL-17 signaling decreased tumor growth and improved the response to anti-cancer therapies.^[30]^

#### 2.8.6. Immunotherapy synergies

Neutrophil-driven inflammation can impede the effectiveness of ICIs, such as anti-PD-1/PD-L1 and anti-CTLA-4 therapies, by creating an immunosuppressive microenvironment. Combining neutrophil-targeted therapies with ICIs could enhance antitumor immunity. For example, chemokine receptor antagonists (such as CXCR2 inhibitors) or TGF-β inhibitors, which reduce neutrophil recruitment and polarization, could complement the action of ICIs by reducing immune suppression within the TME. Studies in various cancers have demonstrated that combining neutrophil-targeted therapies with ICIs improves treatment responses and inhibits tumor progression more effectively than monotherapies.^[43]^

#### 2.8.7. Targeting neutrophil-mediated angiogenesis

Neutrophils contribute to angiogenesis, a critical process for tumor growth and metastasis, by releasing pro-angiogenic factors such as VEGF, MMPs, and IL-8. These factors promote the formation of new blood vessels, which supply oxygen and nutrients to growing tumors. VEGF inhibitors and MMP inhibitors are being explored as adjunctive therapies to target neutrophil-driven angiogenesis in breast cancer. Preclinical studies have shown that targeting angiogenesis through these pathways reduces tumor size and metastasis, highlighting the importance of neutrophils in tumor vascularization. Combining angiogenesis inhibitors with chemotherapy or immunotherapy could improve treatment efficacy by restricting tumor blood supply and reducing metastasis.^[44]^

### 2.9. Therapeutic risks: side effects, off-target immune suppression, and feasibility of neutrophil-targeted strategies

While preclinical findings advocate for neutrophil modulation to curb tumor progression and metastasis, a closer inspection reveals a constellation of concerns – ranging from unintended immunosuppression to questionable clinical feasibility. These concerns warrant careful scrutiny, particularly in light of the delicate balance between tumor control and immune competence.^[40]^ A central challenge lies in the nonselective nature of many neutrophil-targeting agents, which can inadvertently impair host defense mechanisms. Neutrophils play a fundamental role in pathogen clearance, wound healing, and tissue homeostasis. Broad-spectrum depletion strategies – such as CXCR2 inhibitors or anti-Ly6G antibodies – though effective in reducing tumor-associated neutrophils in experimental models, can result in profound neutropenia. This opens the door to opportunistic infections, delayed tissue repair, and systemic inflammatory dysregulation, particularly in immunocompromised patients undergoing chemotherapy. In clinical settings, such risks may outweigh the potential oncologic benefit.^[41]^ Furthermore, the immune system’s redundancy and interconnectedness make selective neutrophil modulation difficult. Inhibiting neutrophil extracellular trap (NET) formation, for instance, via PAD4 inhibitors or DNase therapies, may reduce metastasis but could also interfere with innate immune responses to circulating pathogens. This becomes especially concerning in perioperative or post-chemotherapy patients, where immune suppression could heighten morbidity.^[24]^

Another area of concern is off-target immune suppression, especially when interventions inadvertently affect other myeloid or lymphoid populations. Several agents targeting neutrophil recruitment – such as CXCL8 (IL-8) inhibitors – also influence monocyte and macrophage migration. As a result, the immune milieu may be broadly altered in unintended ways, potentially blunting antitumor T-cell responses or impairing vaccine efficacy. This highlights the need for interventions that are not only potent but also precisely targeted.^[42]^ The feasibility of translating neutrophil-modulatory therapies into the clinic is equally fraught with challenges. Most candidate therapies remain in preclinical or early-phase trials, and long-term safety data are scarce. In addition, the variability of neutrophil phenotypes across patients – driven by tumor subtype, stage, systemic inflammation, and comorbid conditions – complicates patient stratification and response prediction. Without validated biomarkers to guide therapy selection or monitor treatment response, clinical trials risk being underpowered or misdirected.^[30]^ Moreover, therapeutic interventions that rely on systemic delivery routes may fail to reach neutrophils localized within the tumor microenvironment, where they are most active and phenotypically distinct. The development of localized delivery systems, such as nanocarriers or tumor-targeted antibodies, remains technically demanding and expensive, raising questions about accessibility and cost-effectiveness in routine clinical use.^[43]^ Ethical concerns also surface. Given the ambiguous role of neutrophils – capable of both promoting and restraining tumors depending on the context – aggressively targeting them may carry unintended consequences, especially in early-stage disease where their anti-tumoral functions might still be intact. A nuanced approach, such as modulating neutrophil function rather than eliminating them altogether, may offer a safer and more sustainable path, but this requires a deeper understanding of neutrophil plasticity and its regulation within the tumor niche.^[44]^

### 2.10. New perspectives: rethinking neutrophils in breast cancer

The evolving understanding of neutrophils in breast cancer is ushering in a new era of exploration, marked not just by caution against their tumor-promoting activities, but by an appreciation of their plasticity and potential therapeutic value. Once viewed merely as terminally differentiated foot soldiers of the innate immune system, neutrophils are now being reconsidered through a more nuanced lens – one that sees them as dynamic players whose roles are defined by temporal, spatial, and molecular cues.^[45]^ One of the most promising new perspectives is the concept of neutrophil reprogramming rather than depletion. Instead of indiscriminately eliminating neutrophils, researchers are exploring strategies to convert tumor-promoting (N2) phenotypes back to tumor-suppressing (N1) phenotypes. This idea is supported by growing evidence that neutrophil polarization is reversible and influenced by cues from the tumor microenvironment. Therapeutic modulation using agents like interferon-beta, TGF-β inhibitors, or epigenetic modulators may tip the balance in favor of an antitumor immune response, offering a refined and potentially safer intervention strategy.^[46]^ Another forward-thinking approach lies in the integration of single-cell and spatial transcriptomics, which is transforming the way we view the immune landscape of tumors. Rather than relying on bulk analysis, which masks cellular heterogeneity, these technologies allow us to dissect neutrophil subpopulations and their spatial interactions with cancer and stromal cells in situ. This deeper profiling not only clarifies functional roles but also opens doors to the identification of context-specific therapeutic targets, particularly in aggressive breast cancer subtypes like triple-negative breast cancer.^[47]^

Emerging data also highlight the potential of circulating neutrophil signatures as noninvasive biomarkers. Shifts in gene expression profiles, surface markers, or granule protein levels in peripheral blood neutrophils may reflect underlying tumor activity, enabling early detection, real-time monitoring of treatment response, or prediction of metastasis. The development of such “liquid biopsy” approaches could significantly enhance personalized cancer care.^[19]^ Furthermore, there is growing interest in the cross-talk between neutrophils and other immune cells such as T cells, dendritic cells, and natural killer cells. Novel insights suggest that neutrophils do not merely suppress or support these cells but can act as antigen-presenting cells under certain conditions, influencing adaptive immunity in unexpected ways. Harnessing this immunological cross-talk could yield innovative combination therapies that integrate neutrophil modulation with immune checkpoint blockade or cancer vaccines.^[48]^ A particularly exciting frontier involves neutrophil-derived extracellular vesicles. These vesicles, packed with bioactive molecules, can travel systemically and influence distant tissues, including pre-metastatic niches. Their content, which includes microRNAs, enzymes, and cytokines, holds diagnostic potential and may be engineered to deliver therapeutic payloads directly to tumors – blurring the lines between immunomodulation and precision nanomedicine.^[49]^ Patient-specific and subtype-specific approaches are gaining traction. Recognizing that neutrophil activity varies not only between individuals but also between tumor subtypes, researchers are moving toward tailored interventions. For example, neutrophils in hormone receptor-positive breast cancer may differ functionally from those in HER2-positive or basal-like subtypes. A deeper subtype-aware stratification could enhance both treatment efficacy and safety.^[50]^

### 2.11. Controversies, limitations, and potential biases in neutrophil-centric cancer research

The dualistic role of neutrophils in tumor progression remains one of the most controversial areas in cancer immunology, and breast cancer offers a compelling yet convoluted case study. A critical examination of the literature reveals not only scientific uncertainties but also methodological limitations and underlying biases that may shape or distort prevailing narratives. One major source of controversy stems from conflicting interpretations of neutrophil phenotypes. The N1/N2 paradigm, while conceptually useful, is increasingly being challenged for its oversimplification. Many cited studies – including those based on murine models – categorize neutrophils based on a narrow set of markers or cytokine profiles. However, these categorizations may not reflect the full complexity of neutrophil behavior in vivo. Furthermore, human tumors may not exhibit the same clear polarization observed in animal studies, raising concerns about translational relevance. The reliance on animal models, particularly mice with genetically altered immune systems, introduces species-specific biases that limit extrapolation to human disease.^[51]^ Equally problematic is the heterogeneity in defining and measuring the NLR as a prognostic marker. Despite its widespread use, discrepancies in cutoff points, patient populations, and clinical endpoints undermine the consistency of findings. Some studies report a strong association between elevated NLR and poor survival outcomes, while others find no statistically significant correlation. This inconsistency may stem from retrospective designs, limited sample sizes, and a lack of standardized protocols, all of which compromise the robustness of the data. Moreover, many of these studies do not control adequately for confounding variables such as infection, systemic inflammation, or concurrent therapies – each of which can independently influence neutrophil counts and skew results.^[52]^

Another area riddled with controversy is the role of NETs. While NETs have been implicated in fostering metastasis by trapping circulating tumor cells and promoting endothelial dysfunction, the evidence is far from conclusive. A significant number of studies demonstrating NET-mediated tumor progression rely on in vitro assays or xenograft models with artificially induced NET formation. The artificial induction of NETs via pharmacological agents like PMA (phorbol 12-myristate 13-acetate) may not reflect physiological conditions within the tumor microenvironment. Such experimental artifacts could overstate the role of NETs in tumor biology. In addition, the specificity of markers used to identify NETs – such as citrullinated histone H3 or myeloperoxidase-DNA complexes – has been called into question, with concerns about cross-reactivity and lack of validation in clinical samples.^[51]^ The temporal dynamics of neutrophil activity also present a significant interpretive challenge. Some studies suggest that neutrophils exhibit tumor-suppressive properties in early disease stages, only to transition into a pro-tumor phenotype as the tumor evolves. However, this claim is often inferred from cross-sectional data, rather than from true longitudinal studies. This raises the issue of temporal bias – assuming causality based on static snapshots rather than dynamic observations. Without time-resolved data tracking neutrophil function across disease progression, any conclusions about their shifting roles remain speculative. Moreover, publication bias may skew the perceived importance of neutrophils in breast cancer. Studies with dramatic or novel findings – such as neutrophil-mediated metastasis or immunosuppression – are more likely to be published in high-impact journals than those reporting null results or minimal effects. This bias inflates the apparent consensus and may obscure a more nuanced or even contradictory reality. There is also a funding bias in favor of immunotherapy-related research, which may prioritize studies linking neutrophils to immune evasion mechanisms while neglecting other potential functions such as tissue remodeling or angiogenesis.^[52]^ Methodological constraints in clinical studies – such as reliance on bulk RNA sequencing, lack of single-cell resolution, or absence of spatial context – can obscure cell-specific roles and intercellular interactions. Without distinguishing between tumor-infiltrating neutrophils, circulating neutrophils, and neutrophil precursors, researchers risk conflating distinct populations with potentially divergent functions.

### 2.12. Recommendations

#### 2.12.1. Development of neutrophil-specific targeted therapies

A promising approach for treating breast cancer involves the development of therapies that specifically target neutrophils and their pro-tumorigenic functions. Research should focus on designing CXCR2 inhibitors, IL-17 inhibitors, and TGF-β blockers to prevent neutrophil recruitment and polarization. Preclinical studies and clinical trials must continue to evaluate the efficacy and safety of these targeted agents in combination with standard therapies like chemotherapy, immunotherapy, or anti-angiogenic treatments. Targeting neutrophils at various stages of their activation and recruitment could greatly enhance cancer immunotherapies.

#### 2.12.2. Combination therapies with immunotherapies and chemotherapies

Neutrophils often contribute to resistance against conventional therapies, such as chemotherapy and immunotherapy, by promoting tumor-promoting inflammation and immune suppression. Combining neutrophil-targeted therapies with ICIs, chemotherapy, and angiogenesis inhibitors should be explored to break the immunosuppressive TME and improve patient responses. Clinical trials should investigate the synergistic effects of these combinations to ensure enhanced tumor control while minimizing adverse effects.

#### 2.12.3. Personalized approaches to neutrophil targeting

As neutrophil behavior can vary across patients and even between tumor types, personalized medicine should be adopted in strategies targeting neutrophil-driven inflammation. By using molecular profiling of patients’ tumors, it will be possible to identify specific inflammatory mediators and neutrophil-related biomarkers that predict treatment outcomes. Personalized treatments could involve the use of biomarkers to select patients who are more likely to benefit from neutrophil-targeting therapies, optimizing therapeutic responses.

#### 2.12.4. Long-term monitoring and safety considerations

Long-term safety monitoring is essential when targeting neutrophils, as neutrophils are crucial for immune defense and tissue repair. Close surveillance for infection risks, potential immunosuppression, and neutropenia in patients undergoing neutrophil-targeted therapies will be critical to ensure that the benefits of treatment outweigh potential harm. Furthermore, adaptive monitoring techniques should be implemented to adjust therapeutic regimens based on patient response and immune status.

#### 2.12.5. Focus on neutrophil-derived extracellular traps

Given that NETs contribute to metastasis and immune evasion in breast cancer, it is recommended that therapies be developed that specifically target the formation and activity of NETs. Strategies that inhibit NETosis, such as the use of DNase I, histone deacetylase inhibitors, or antioxidants, should be further studied. These agents could potentially reduce tumor spread and improve responses to conventional therapies by decreasing the scaffold that facilitates metastasis.

#### 2.12.6. Enhancing neutrophil’s tumor-suppressive roles

Neutrophils are not always detrimental to the tumor environment, as they can also act in antitumor roles. Future research should investigate methods to polarize neutrophils into N1 phenotypes, which promote antitumor immunity. Investigating the molecular pathways involved in neutrophil polarization and exploring the potential of pharmacological agents that stimulate N1 neutrophils could provide new therapeutic opportunities in breast cancer treatment.

#### 2.12.7. Preclinical models to simulate neutrophil interaction

More advanced preclinical models, including humanized mouse models and tumor organoids, should be developed to more accurately replicate neutrophil-tumor interactions. These models could enable researchers to evaluate neutrophil-driven inflammation and test neutrophil-targeted therapies in a more clinically relevant context. Improved models are crucial for understanding the complexities of neutrophil function in cancer progression and the TME.

#### 2.12.8. Collaboration and multidisciplinary research

To better address the complexities of neutrophil-driven inflammation in cancer, multidisciplinary collaborations among immunologists, oncologists, pharmacologists, and biologists are necessary. Collaborative efforts should focus on the identification of novel neutrophil-targeting agents, the development of combination treatment strategies, and the improvement of preclinical models. Such collaborations will accelerate the translation of research into clinical applications and improve therapeutic outcomes in breast cancer.

## 3. Conclusion

Neutrophils play a complex and dual role in breast cancer, contributing to both tumor progression and immune defense. Their involvement in tumor inflammation is primarily driven by key pathways such as the CXCL8/CXCR2 axis, NF-κB signaling, ROS production, and the formation of NETs. While neutrophils can promote tumor growth, angiogenesis, and metastasis, they can also participate in antitumor immune responses under certain conditions. Targeting neutrophil-driven inflammation, therefore, presents a promising therapeutic strategy to modulate the tumor microenvironment and enhance the effectiveness of current treatments.

## Author contributions

**Conceptualization:** Emmanuel Ifeanyi Obeagu.

**Methodology:** Emmanuel Ifeanyi Obeagu.

**Resources:** Emmanuel Ifeanyi Obeagu.

**Supervision:** Emmanuel Ifeanyi Obeagu.

**Validation:** Emmanuel Ifeanyi Obeagu.

**Visualization:** Emmanuel Ifeanyi Obeagu.

**Writing** – **original draft:** Emmanuel Ifeanyi Obeagu.

**Writing** – **review & editing:** Emmanuel Ifeanyi Obeagu.
